# *nr3c1* null mutant zebrafish are viable and reveal DNA-binding-independent activities of the glucocorticoid receptor

**DOI:** 10.1038/s41598-017-04535-6

**Published:** 2017-06-29

**Authors:** N. Facchinello, T. Skobo, G. Meneghetti, E. Colletti, A. Dinarello, N. Tiso, R. Costa, G. Gioacchini, O. Carnevali, F. Argenton, L. Colombo, L. Dalla Valle

**Affiliations:** 1 Department of Biology, University of Padova, Padova, Italy; 2 Department of Molecular Medicine, University of Padova, Padova, Italy; 3 Department of Life and Environmental Sciences, Marche Polytechnic University, Ancona, Italy

## Abstract

Glucocorticoids (GCs) play important roles in developmental and physiological processes through the transcriptional activity of their cognate receptor (Gr). Using CRISPR/Cas9 technology, we established a zebrafish null Gr mutant line and compared its phenotypes with wild type and a zebrafish line with partially silenced *gr* (*gr*^*s357*/*s357*^). Homozygous *gr*^−/−^ larvae are morphologically inconspicuous and, in contrast to *GR*^−/−^ knockout mice, viable through adulthood, although with reduced fitness and early life survival. Mutants *gr*^−/−^ are fertile, but their reproductive capabilities fall at around 10 months of age, when, together with cardiac and intestinal abnormalities already visible at earlier stages, increased fat deposits are also observed. Mutants show higher levels of whole-body cortisol associated with overstimulated basal levels of *crh* and *pomca* transcripts along the HPI axis, which is unresponsive to a mechanical stressor. Transcriptional activity linked to immune response is also hampered in the *gr*^−/−^ line: after intestinal damage by dextran sodium sulphate exposure, there are neither inflammatory nor anti-inflammatory cytokine gene responses, substantiating the hypothesis of a dual-action of the GC-GR complex on the immune system. Hence, the zebrafish *gr* mutant line appears as a useful tool to investigate Gr functions in an integrated *in vivo* model.

## Introduction

Glucocorticoids (GCs), predominantly cortisol in humans and teleost fish, and corticosterone in rodents, are steroid hormones secreted by the adrenal cortex in mammals and the interrenal tissue of the head kidney in teleosts. These hormones regulate many physiological processes, including glucose homeostasis, intermediary metabolism^1^, inflammatory^2^ and stress responses. Moreover, in fish, GCs are also involved in water and electrolyte homeostasis^3^ and their production is regulated by the hypothalamus-pituitary-interrenal (HPI) axis that is equivalent to the mammalian hypothalamus-pituitary-adrenal (HPA) axis^4,5^.

In agreement with the pleiotropic effects of GCs, their cognate receptor is ubiquitously expressed, according to specific tissue/cell type regulation^6^.

In mammals, the activation of the GC signalling pathway depends mainly on the binding to the cognate cytoplasmic GC receptor, GR, a member of the nuclear receptor family of ligand-activated transcription factors that regulates tissue-specific sets of genes. After GC binding, GR translocates into the nucleus, where it directly binds to GC responsive elements (GREs) in the promoter region of target genes, thus regulating their transcription in positive or negative (nGREs) ways. In addition, the complex hormone-GR may indirectly inhibit transcription, in particular of pro-inflammatory genes, by means of protein-protein interactions with transcription factors, such as AP-1 and NF-kB or other DNA-binding proteins^7^.

Most teleosts present two *gr* genes, called *gr1* and *gr2*^8^ but in the zebrafish (*Danio rerio*) model organism, the genome contains a single *gr* gene^9^, the structure of which is highly similar to the organization of the human gene, *hGR*^10^. In humans, two main protein isoforms, GRα and GRβ, are produced by means of alternative splicing processes. Although the splicing events are different in the two species, the zebrafish Grβ is comparable to its human equivalent in structure and expression level^11^, but was lately found to differ in function, because it does not act as a dominant-negative inhibitor of zGrβ in either cultured cells or zebrafish larvae^12^.

Moreover, alternative translation initiation from *hGR* mRNA can produce eight translational isoforms that share both the capability to bind to DNA and the ligand affinity due to the presence of the ligand-binding domain (LBD)^13^. However, these isoforms differ in subcellular localization and the ability to regulate gene transcription^14,15^. To study GC functions, GR-null mice have been generated, but they undergo perinatal mortality due to defects in lung maturation and cannot be used to analyse complete *GR* gene silencing beyond this stage^16^. This problem does not concern mice carrying the *GR*^*dim*/*dim*^ mutation, since they are normal and viable. In these mutants, GRE-dependent gene transcription is absent because GR homodimerization and DNA binding is impaired, but other DNA-binding-independent activities of GR, such as the cross-talk with other transcription factors, are allowed^17^.

In the zebrafish model, morpholino knockdown of maternally derived *gr* mRNA triggers several profound developmental defects that limit survival at the larval stage^18,19^. Complete *gr* gene silencing is also likely not attained in an adult viable mutant zebrafish strain, named *gr*^*s357*/*s357*^, in which a single base-pair mutation in the DNA-binding domain (DBD) disrupts GRE-dependent activity, but possibly not protein-protein interactions^20^.

To better understand all activities of the GC-Gr complex, we have generated a zebrafish Gr-KO line using CRISPR/Cas9 technology. Although the survival rate at post-larval stages is markedly reduced, the line is viable and fertile. Our results indicate that homozygous mutants display physiological responses clearly linked to GC-resistance, such as overstimulation of basal HPI axis associated with unresponsiveness to a prolonged mechanical stressor. Moreover, they confirm the dual-action model of the GC-GR complex in the immune sequential response with both pro-inflammatory and anti-inflammatory effects, as proposed by Busillo and Cidlowski^21^.

By comparison with *gr*^*s357*/*s357*^ mutants, the Gr-KO line evidences a wider range of suppression of Gr-controlled transcriptional mechanisms on the basis of differential targeting of genes and phenotyping characterisation at the larval and adult stages. This comparative approach provides a new opportunity for discriminating the details of non-canonical Gr activities mediated by protein-protein interactions.

## Results

### Generation and characterization of a stable zebrafish *gr* mutant line with CRISPR/Cas9

**Figure 1:**
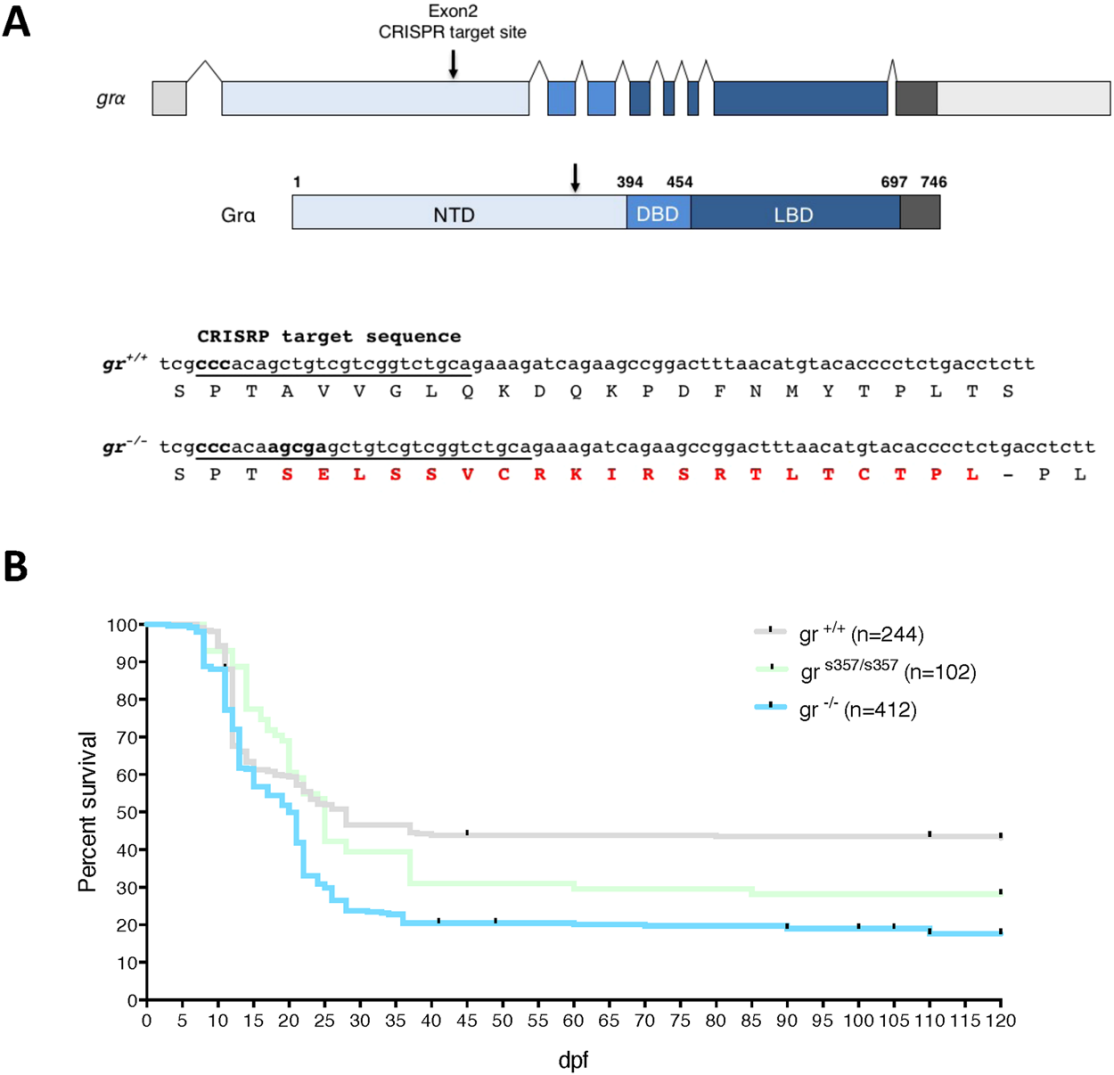
Inactivation of the zebrafish *gr* gene and survival analysis of *gr* mutants. (**A**) Schematic representation of the zebrafish *gr* gene. Exons are shown as boxes with untranslated regions in light grey. Arrow shows the position of the CRISPR targeted site in exon 2 of the gene structure and with respect to DBD and LBD in the protein structure. In the *gr*^+/+^ and *gr*^−/−^ sequences, the CRISPR targeted site is underlined and the protospacer-adjacent motif (PAM) sequence is labelled in bold. The 5-nucleotide insertion, revealed by sequencing, and the consequent 20 new aa are shown in red and bold. (**B**) Kaplan-Meier survival curves of the three zebrafish genotypes analysed in this study. Time is shown in days. n = number of fish per genotype. The Log Rank test was used for statistical analysis. *gr*^−/−^ vs. *gr*^*s357*/*s357*^: P < 0.006; *gr*^−/−^ vs. *gr*^+/+^: P < 0.0001.

To generate a zebrafish model completely devoid of transcriptional GC activity, we mutated the zebrafish *gr* gene (*nr3c1*) using a CRISPR/Cas9 approach. The line, *nr3c1*^*ia30*/*ia30*^, will be called *gr*^−/−^ hereafter. A heterozygous F1 offspring with a 5-nucleotide insertion in exon 2 was selected and used to obtain the F2 generation. The 5-nucleotide insertion resulted in a frameshift mutation that leads to a premature stop codon located upstream of DBD. The putative encoded protein contains 331 aa (the first 301 of the zebrafish Gr plus 20 new aa), but lacks the domains required for DNA and ligand binding (Fig. 1, panel A).

Quantitative RT-PCR (qRT-PCR) of 5-dpf (days post-fertilization) homozygous mutant larvae showed a statistically significant reduction of *gr* transcript levels to less than 7% of those measured in WT fish (Supplemental Fig. 1, panel A). This result is consistent with nonsense-mediated mRNA decay (NMD) due to premature stop codon^22^, suggesting that the mutation might result in the loss of Gr function. No significant changes of *gr* transcript levels were instead present in *gr*^*s357*/*s357*^ homozygous larvae (Supplemental Fig. 1, panel A).

The mutated region in detectable *gr* transcripts was analysed by RT-PCR and sequencing, confirming the presence of the mutation in the remaining *gr* transcripts of homozygous larvae. The latter, obtained by heterozygous mating, were phenotypically similar to control siblings and segregated according to a Mendelian ratio. Furthermore, they displayed an impaired Visual Background Adaptation **(**VBA) assay, a GC/GRE-dependent neuroendocrine response^23^, that allows visual selection of homozygous larvae (Supplemental Fig. 1, panel B). Genotypes of *gr*^+/+^, *gr*^+/−^ and *gr*^−/−^ samples were confirmed by genomic DNA PCR and agarose gel analysis as well as sequencing of the mutated region (Supplemental Fig. 1, panel C). VBA assessment and genotyping were also used to identify and confirm *gr*^*s357*/*s357*^ homozygous larvae, obtained by heterozygous mating, used in this work.

Gr proteins were analysed by Western blotting of WT and *gr* mutant adult liver proteins (Supplemental Fig. 1, panel D) using an antibody previously shown to bind to zebrafish Gr^3,24^. A band corresponding to the Gr protein was detected in WT, but not in *gr*^−/−^ mutant liver proteins.

Additionally, offspring from incrossed homozygous *gr* mutants raised to adulthood were also viable and fertile, thus excluding that survival of homozygous fish derived from heterozygous females was essentially due to the compensative effects of maternal *gr* transcripts or proteins. Thus, differently from GR-null mice, zebrafish *gr*^−/−^ mutants obtained from heterozygous as well as homozygous carriers survive and reach sexual maturity. However, they are unable to respond to stress stimuli, including the required increment of cortisol levels linked to energy mobilization for gamete production and release in fish^25,26^, a likely cause of their death sometimes shortly after mating.

To analyse this aspect, we examined fish survival during 4 months and obtained Kaplan-Meier survival curves for the three genotypes, *gr*^+/+^, *gr*^−/−^ and *gr*^*s357*/*s357*^, maintained under the same laboratory conditions. Up to 10 dpf, there was no difference in survival between both types of mutants and WT fish (Fig. 1, panel B). The critical rearing period was found for all three genotypes between 10 and 25 dpf. Thereafter, the decline in the survival rate levelled at around 50% in WT fish and 30% in *gr*^*s357*/*s357*^ ones (Fig. 1, panel B), while only 15% of *gr*^−/−^ larvae reached the 4 months of age; this decrease in early life survival was statistically significant compared with *gr*^+/+^ and *gr*^*s357*/*s357*^ genotypes (Fig. 1, panel B).

**Figure 2:**
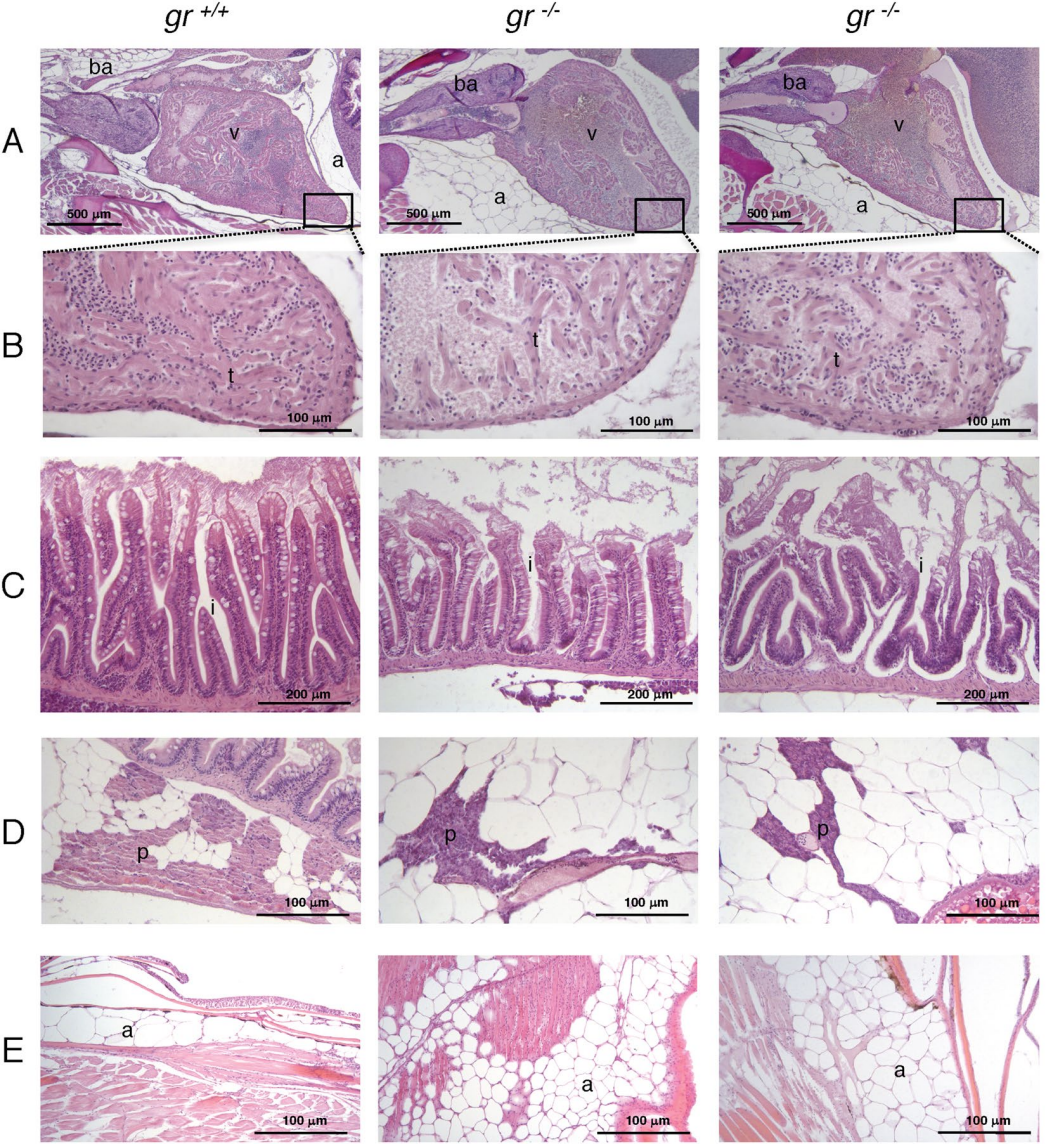
Histology of 8-month-old *gr*^−/−^ and *gr*^+/+^ zebrafish. All histological images of 8-month-old *gr*^−/−^ and *gr*^+/+^ zebrafish were taken from longitudinal sections stained with haematoxylin and eosin (H&E). The left panels represent wild type fish, whereas the middle and right panels show tissues from two mutant fish. (**A**) heart; (**B**) particular of the heart at higher magnification showing reduced trabecular network in the mutant samples; (**C**) intestinal mucosa with sloughing epithelium at the villous tips and reduced height of villi in mutants; (**D**) visceral view showing reduced extension of pancreas in mutants; (**E**) consistent increase of subcutaneous adipose tissue in mutants. ba = bulbus arteriosus; v = ventricle; a = adipose tissue; i = intestine; p = pancreas; t = trabeculae.

Comparative morphological analysis of three 45-day-old *gr*^+/+^, *gr*^−/−^ and *gr*^*s357*/*s357*^ did not reveal any evident histological abnormality in both mutants with respect to WT fish (Supplemental Fig. 2a,b and c), except for the proximal anterior intestine (often referred to as the intestinal bulb) of *gr*^−/−^, in which the epithelium is relatively thinner and flatter in comparison with *gr*^+/+^ and *gr*^*s357*/*s357*^ (Supplemental Fig. 2b), and for the heart ventricle that presented a reduced trabecular network (Supplemental Fig. 2a). Moreover, histological analysis of two 8-month-old *gr*^+/+^ and four *gr*^−/−^ confirmed the morphological alterations in the heart and intestine with a seemingly reduction of the pancreatic tissue (Fig. 2). There was also a clear and consistent accumulation of adipose tissue, in both male and female samples (Fig. 2; Supplemental Fig. 3). Moreover, a 11% reduced heart rate was measured in *gr*^−/−^ larvae (Supplemental Fig. 4).

### Analysis of the GRE-dependent transcription by means of transgenic Tg(9xGCRE-HSV.Ul23:EGFP)^ia20^ background and qRT-PCR analysis

To analyse GRE-dependent transcriptional activity *in vivo*, we crossed the two mutant lines with a GC-responsive zebrafish transgenic line (ia20), in which the enhanced green fluorescent protein (EGFP) transgene is located downstream of nine tandem GRE repeats, thus allowing analysis of endogenous GC- and dexamethasone ﻿(DEX)-induced transcriptional activity by means of fluorescence observation^27^.

GRE-GFP transgenic heterozygous (*gr*^+/−^ and *gr*^*+*/*s357*^) 5-dpf larvae showed a fluorescence decrease of endogenous GC response with respect to *gr*^+/+^ age-matched control siblings (data not shown).

**Figure 3:**
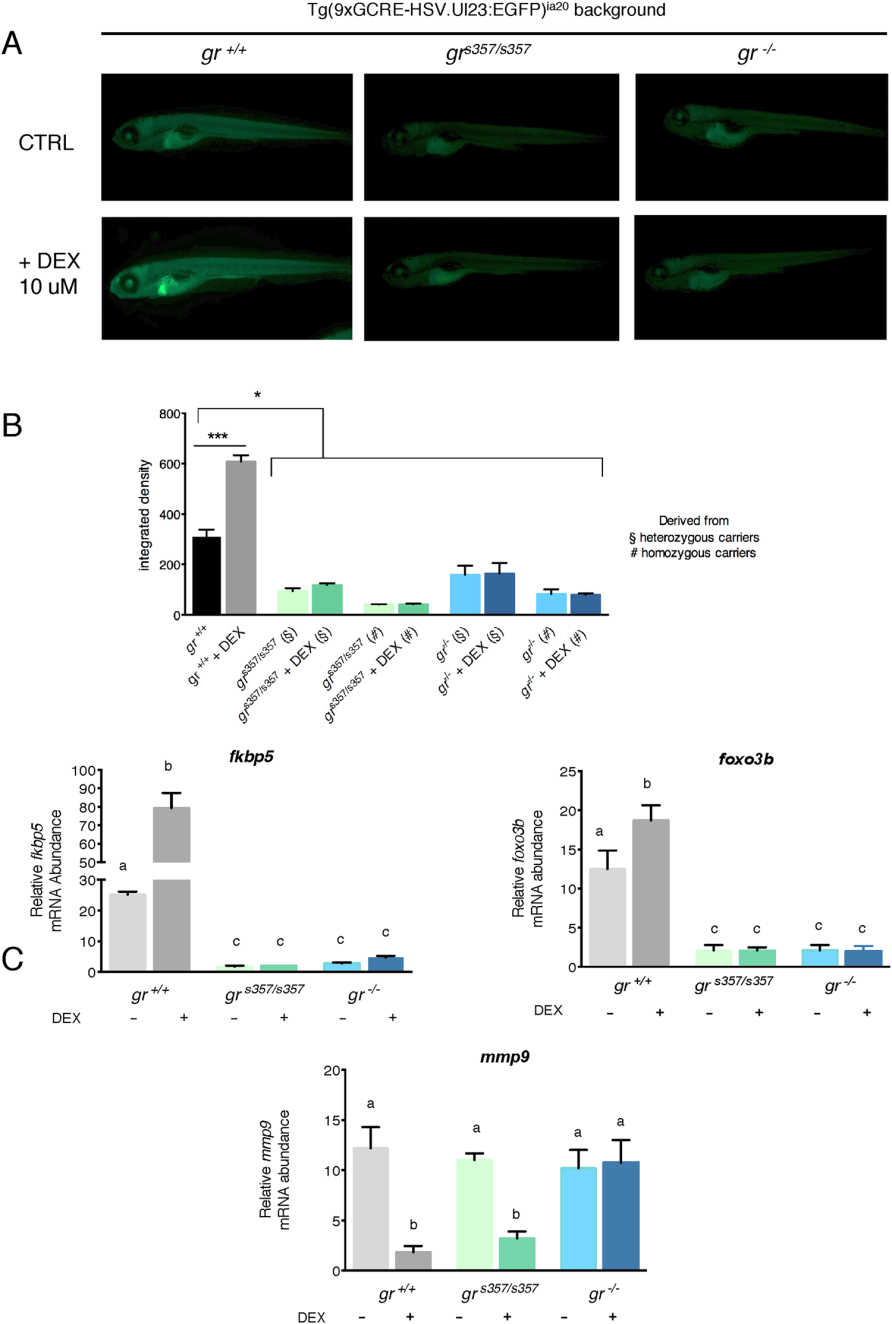
*gr*^*s357*/*s357*^ and *gr*^−/−^ 5-dpf zebrafish larvae in transgenic Tg(9xGCRE-HSV.Ul23:EGFP) background cannot respond to DEX treatment. (**A**) Fluorescence microscopy images of *gr*^+/+^, *gr*^*s357*/*s357*^ and *gr*^−/−^ 5-dpf zebrafish larvae in transgenic Tg(9xGCRE-HSV.Ul23:EGFP)ia20 background. Treated larvae were subjected to 10 μM DEX treatment for 24 h (from 4 dpf to 5 dpf). (**B**) Integrated density analysis of fluorescence of 5-dpf zebrafish larvae of the three genotypes with or without DEX treatment. Homozygous zebrafish mutants deriving from heterozygous or homozygous parents were analysed separately. Values represent the mean ± SEM. Asterisks indicate that expression levels are significantly different from the control (two-way-ANOVA, *P < 0.05, ***P < 0.001). n = 15 larvae for each group. (**C**) qRT-PCR analysis of *fkbp5*, *foxo3b* and *mmp*-*9* in *gr*^+/+^, *gr*^*s357*/*s357*^ and *gr*^−/−^ 5-dpf zebrafish larvae deriving from homozygous parents with or without DEX treatment. Values represent the mean ± SEM. Different letters indicate statistically significant differences checked by two-way ANOVA followed by Tukey’s multiple-comparison test. *fkbp5* (P < 0.01); *foxo3b* and *mmp*-*9* (P < 0.001). Data were generated from four biological replicates.

Moreover, fluorescence levels related to the same response were very low and statistically different from *gr*^+/+^ larvae in homozygous *gr* mutants derived from heterozygous carriers and even lesser in larvae obtained from homozygous carriers (Fig. 3, panel B), likely due to the lack of maternal *gr* transcripts and proteins. After 24 h of 10 μM DEX treatment, a fluorescence increase due to GRE-mediated GC activity was evident and statistically significant only in *gr*^+/+^ larvae. In contrast, in both mutants, fluorescence levels did not show any variation (Fig. 3, panel A and B).

Moreover, the qPCR analysis of *fk506*-*binding protein*-*5* (*fkbp5*), a GRE-regulated gene, demonstrated that it was much less expressed in both mutant larvae than in *gr*^+/+^ and did not show any induction after DEX treatment, while an increase of *fkbp5* gene expression was instead statistically significant in *gr*^+/+^ (Fig. 3, panel C). A similar result was obtained analysing *foxo3b* transcription, thus confirming that also this gene is GRE-regulated.

Analysis of *mmp*-*9*, a metalloproteinase gene known to be down-regulated by GCs^2,28,29^, showed a statistically significant reduction of its expression after DEX in *gr*^+/+^ and *gr*^*s357*/*s357*^ line, but not in *gr*^−/−^, in agreement with the loss of non-canonical, GRE-independent activity.

### The HPI axis is dysregulated in the *gr* mutant lines

**Figure 4:**
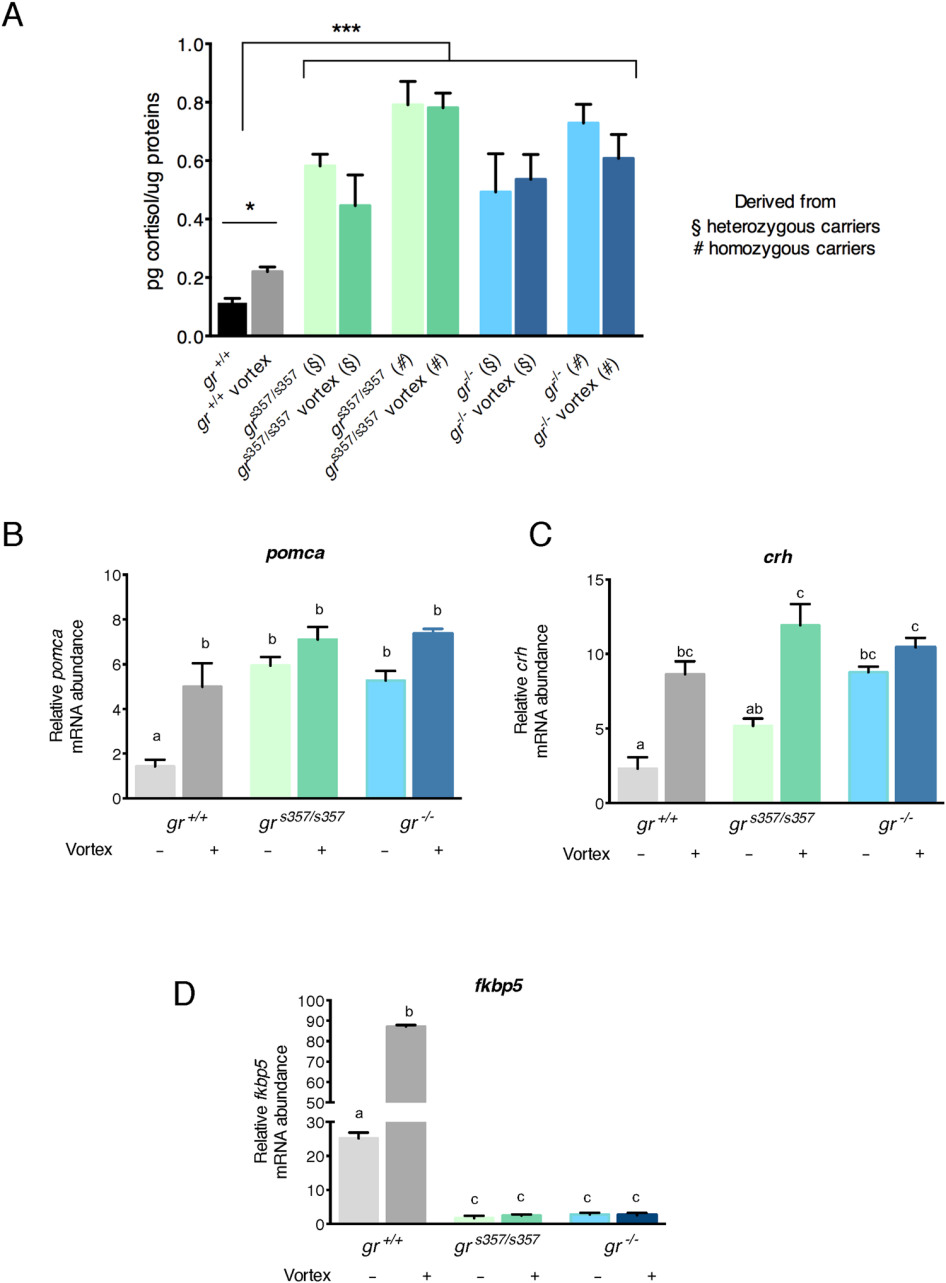
Dysregulation of the HPI axis in the *gr* mutant lines. (**A**) Whole-body cortisol concentrations as determined by RIA in *gr*^+/+^, *gr*^*s357*/*s357*^ and *gr*^−/−^ 5-dpf zebrafish larvae under basal and stressful conditions. Homozygous zebrafish mutants deriving from heterozygous or homozygous parents were analysed separately. Concentrations were determined from 6 pools of 10 larvae (three different experiments) and are expressed as picograms per μg protein (mean ± SEM). Asterisks indicate that the cortisol concentration is significantly different from the control (two-way-ANOVA, *P < 0.05, ***P < 0.001). (**B**) qRT-PCR analysis of *pomca*, *crh* and *fkbp5* in *gr*^+/+^, *gr*^*s357*/*s357*^ and *gr*^−/−^ 5-dpf zebrafish larvae deriving from homozygous parents under basal and stressful conditions. Values represent the mean ± SEM. Different letters indicate statistically significant differences checked by two-way ANOVA followed by Tukey’s multiple-comparison test. *pomca* and *crh* (P < 0.01); *fkbp5* (P < 0.001). Data were generated from four biological replicates.

Whole-body cortisol, as measured by RIA in 5-dpf larvae, showed statistically significant higher levels in both mutant lines compared to *gr*^+/+^ fish. Moreover, in mutant fish, cortisol level did not show any change after mechanical stress, when compared with age-matched controls (Fig. 4, panel A).

**Figure 5:**
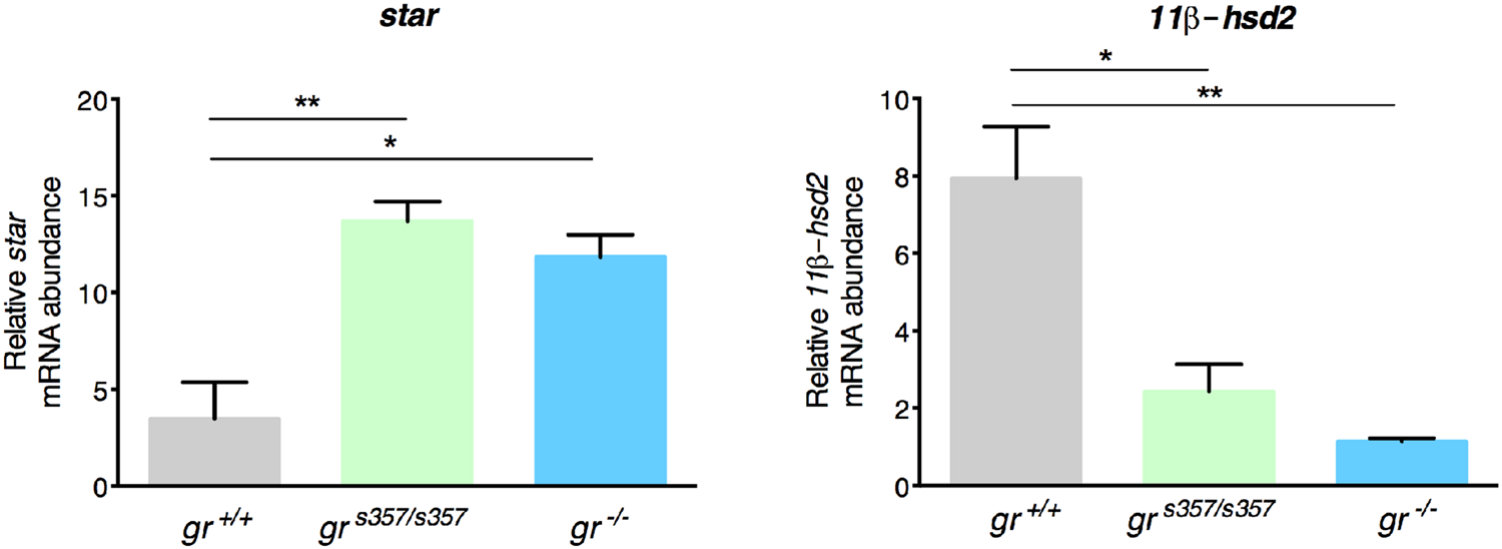
Expression of *star* and *11β*-*hsd type 2* genes supports cortisol synthesis in *gr* mutant lines. qRT-PCR analysis of *star* and *11β*-*hsd type 2* in *gr*^+/+^, *gr*^*s357*/*s357*^ and *gr*^−/−^ 5-dpf zebrafish larvae deriving from homozygous parents under basal conditions. Values represent the mean ± SEM. Asterisks indicate that expression levels are significantly different from the control (one-way ANOVA; *P < 0.05, **P < 0.01). Data were generated from four biological replicates.

Disruption of the negative HPI axis feedback during the stress response was also demonstrated by qPCR that measured higher levels of *pomca* basal transcripts in mutant larvae of both genotypes. After stress induction, *pomca* transcripts showed a statistically significant increase in *gr*^+/+^ fish, but no significant changes were measured in the two mutant lines (Fig. 4, panel B). A similar result was obtained with *crh* mRNA abundance in the *gr*^−/−^ line before and after stress induction. However, basal levels of *crh* transcripts in the *gr*^*s357*/*s357*^ line, although higher, were not statistically different from those of *gr*^+/+^, and showed a clear and statistically significant increase after stress induction (Fig. 4, panel C). Stress induction did not determine any variation in the *fkbp5* expression of both mutant lines (Fig. 4, panel D). Finally, to confirm the perturbation of the HPI axis, we also measured the basal levels of *star* and *11β*-*hsd type 2* gene transcripts and found the first up-regulated and the second down-regulated in both mutants (Fig. 5).

### Analysis of the anti-inflammatory response in *gr* mutant lines

In order to study the GRE-independentcross-talk of Gr with other pathways, we analysed the response to an inflammatory stimulus in the two *gr* mutant lines by qRT-PCR expression analysis of genes involved in immune reaction as compared to WT fish.

**Figure 6:**
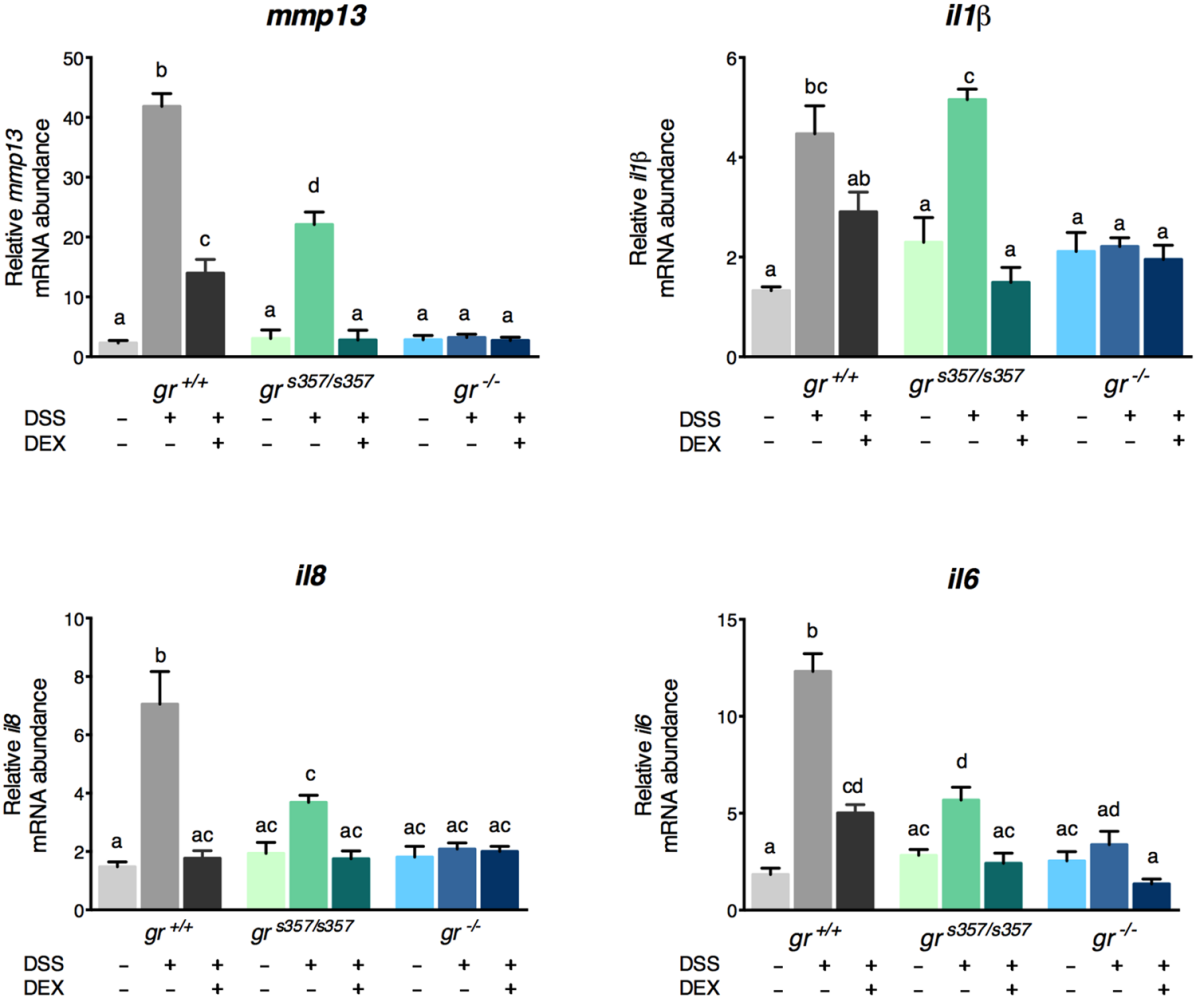
The *gr*^−/−^ mutant line does not respond to an inflammatory stimulus. qRT-PCR analysis of *mmp*-*13*, *il1β*, *il8* and *il6* in *gr*^+/+^, *gr*^*s357*/*s357*^ and *gr*^*−/−*^ 5-dpf zebrafish larvae deriving from homozygous parents under basal conditions and after a 20-h DSS treatment followed or not by 24-h DEX treatment. Values represent the mean ± SEM. Different letters indicate statistically significant differences checked by two-way ANOVA followed by Tukey’s multiple-comparison test. *mmp13* (P < 0.001), *il1β* (P < 0.01); *il8* and *il6 (*P < 0.001). Data were generated from four biological replicates.

In control 5-dpf larvae of all genotypes, transcription levels of *mmp*-*13* were very low but, after dextran sodium sulphate (DSS) treatment, they showed up to 40- and 20-fold induction in *gr*^+/+^ and *gr*^*s357*/*s357*^, respectively. No increase was instead observed in the *gr*^−/−^ line. After DEX treatment, *mmp*-*13* expression returned to basal level (*gr*^*s357*/*s357*^) or was statistically reduced (*gr*^+/+^) (Fig. 6).

Similar results were obtained with *il1β*, *il8 and il6* genes. In each case, DSS treatment did not up-regulate the expression of cytokines linked to the inflammatory process in the *gr*^−/−^ line and DEX treatment resulted in no expression changes (Fig. 6). Longer DSS treatments could not be used due to a mortality increase in *gr* mutant larvae, particularly of the *gr*^−/−^ line (data not shown). Finally, the mucin produced by Goblet cells, which is commonly increased during an active inflammation state^30^, was quantified in the mid-distal intestine of larvae by Alcian blue staining (Supplemental Fig. 5). After DEX treatment, the higher levels of mucin induced by DSS treatment were reduced in *gr*^+/+^ and *gr*^*s357*/*s357*^. Notably, no statistically significant variations were measured in *gr*^−/−^ zebrafish (Supplemental Fig. 5).

## Discussion

### Divergence in GRE-independent target gene transcription in the two mutant lines

We have generated a viable and fertile zebrafish line bearing a homozygous mutation in the *gr* gene in order to suppress the multiple transcriptional responses to the GC-Gr complex. This was achieved by CRISPR/Cas9 insertion of a 5-nucleotide segment in exon 2 (first coding exon) of the *gr* gene in a strain harbouring a transgene with GRE-dependent GFP-expression^27^.This resulted in a frame-shift which led to a premature stop codon, thus blocking translation upstream of DBD, LBD and AF2 domains of the corresponding protein.

Fluorescence unresponsiveness, even after DEX exposure, provided an initial clue about the effectiveness of the mutation in preventing activation of GFP expression. Such insensitivity to both endogenous and exogenous GCs was more marked in homozygous mutant larvae from homozygous carriers than heterozygous ones, analogously to the *gr*^s357/s357^ mutant strain with compromised DBD codification alone, used for comparison together with WT fish. Similarly to the GRE-responsive transgene, GRE-mediated transcription of the endogenous genes *fkbp5* and *foxo3b* was also blunted as basal levels and after DEX treatment in both mutants.

Conversely, other target genes that were sensitive to GCs in the *gr*^s357/s357^ mutants were found to be unaffected in the *gr*^−/−^ line due to missing Gr protein, including transactivation domains required for protein-protein interactions with other transcription factors or components of other regulatory pathways. Divergent transcriptomic responses were observed with DEX suppression of the DSS-induced transcription of the cytokine genes *il1β*, *il8* and *il6* and of the metalloproteinase genes *mmp*-*13* and DSS-unchallenged *mmp-9* in *gr*^*s357/s357*^ mutants, but not in the other ones. Transcriptional activity of *gr*^*s357/s357*^ mutant line was *in vitro* analysed by Ziv and co-workers (2013)^31^ that found lack of Gr genomic activity including transcriptional repression linked to AP1 or NF-kB transcription factors. However, these data are not in conflict with our definition of *gr*^*s357/s357*^ as being partially silenced: Gr can regulate gene repression either by binding directly to a GRE and interacting with other transcription factors^32^ (and this way is lost also in *gr*^*s357/s357*^) or by tethering with other DNA binding proteins, a way lost in the gr^−/−^ null line while possibly retained in the *gr*^*s357/s357*^ mutant. Hence, the *gr*^*s357/s357*^ line directly reveals GRE-independent GC-GR transcribing activities due to their persistence, while the gr^−/−^ line indirectly confirms their occurrence due to their absence with consequent effects on the phenotype, as discussed in details below.

### Evaluation of *gr* silencing in the *gr*^−/−^ mutant line

While these effects point to marked GC resistance in *gr*^−/−^ mutants, minor interference with full Gr silencing at the translation step still must be considered. The fact that the overall *gr* transcript level was reduced by 93% in *gr*^−/−^ mutants compared to WT, indicates that the nonsense-mediated mRNA decay (NMD) machinery, a surveillance pathway that prevents truncated protein formation^33^, was highly efficient, though not completely, as attested by the positive result of qRT-PCR analysis with primers designed on the C-terminal region. The remaining *gr* mRNA can be translated due to the occurrence of four alternative translation-initiation sites (AUG) downstream of the premature stop codon. Two of them are located just downstream of the premature stop codon, allowing translation of a shorter but functional Gr. However, the so-called Kozak sequences of these AUGs do not correspond to those with high translational efficiency, as reported by a zebrafish transcriptome analysis^34^.

Considering that only 7% of *gr* mRNA was left for functional translation and that the translation efficiency of the two sites is rather low, a minimal amount of active Gr is expected, as confirmed by the negative response of the antibody detection assay for zGrα in *gr*^−/−^ mutants compared to controls. Furthermore, the receptor would have a truncated N-terminal domain resembling the human GR-D subtypes with more or less equally shortened exon 2 due to alternative start sites. These subtypes generally showed lower transcriptional efficiency on target genes with respect to the hGRα-A, the classical full-length hGR protein^35,36^. The remaining two start sites in zebrafish are confined towards the end of the DBD coding region and would translate a protein capable of binding at most to the ligand, but not directly to DNA. Thus, we may assume that Gr silencing is functionally complete with likely negligible interference by Gr activity.

### Comparison between *gr* knockout mutants and *gr* knockdown morphants

The main advantages of exploring Gr regulatory role in zebrafish knockout mutants with respect to the use of morpholino oligos to knockdown early *gr* mRNA translation or manipulation of cortisol content in newly-fertilized embryos are that no exogenous substance is introduced by microinjection, the experimental condition persists throughout adult life and can be genetically transmitted by homozygous parents.

It should be noticed that developmental defects in *gr* morphant phenotypes were much more severe than those observed in *gr*^−/−^ mutants and were mostly incompatible with survival beyond the larval stage. Two independent studies have reported profound morphological abnormalities in morphant embryos and larvae of zebrafish encompassing reduced growth, abnormal mesoderm formation with altered somitogenesis, craniofacial and caudal deformities, and malformations of neural, cardiac, vascular and visceral organs^18,19^. However, less serious malformations and viability to adulthood were documented by Wilson and coworkers^37^ in fish subjected to a dosage of *gr*-ATG morpholino lower than those used by the above authors^18,19^. Other laboratories have also found differences between genetic null-mutants and knockdown morphants, which are generally more critically affected. While these differences could be partially ascribed to off-target/toxic effects of MOs, it has been proposed that, in mutant lines, the effects of deleterious mutations could be contrasted by the activation of compensatory pathways with altered gene expression of unknown genes^38^. In the future, a transcriptome analysis will help to explore this possibility.

### Phenotyping differences between mutant lines

Despite their inconspicuous external morphology, early life survival was indeed markedly curtailed in *gr*^−/−^ mutants not only in comparison with WT controls, but also with the *gr*^s357/s357^ mutant line, implying a deeper abrogation of indispensable Gr functions. In particular, preliminary phenotyping disclosed an imperfect histological architecture in the anterior intestine. The fact that in *gr*^−/−^ adults the bulb epithelium was rather thin and less elaborated into folds than in WT and *gr*^s357/s357^ mutants suggests lesser digestive capacity and nutrient absorption, traits unfavourable as to life expectancy. Another detrimental trait was the reduced trabecular network of the heart ventricle indicating weaker propulsive force in blood circulation, also confirmed by the reduced heart rate measured in *gr*^−/−^ larvae. Interestingly, a cardiac phenotype very similar to the mutant one was obtained by Wilson and coworkers^37^ in adults subjected to *gr*-ATG morpholino.

Actually, both intestine and heart are well-known GC targets, as demonstrated by the strong EGFP fluorescence in response to endogenous GCs or DEX treatment of the transgenic zebrafish line ia20^27^.

Histological analysis of 8-month-old mutants revealed also a significant increase of subcutaneous adipose tissue. More extensive interrogation of Gr functions may expose other defects in *gr*^−/−^ organogenesis, thus explaining the steeper decline of survival between 10 and 25 dpf.

### Disruption of HPI function and stress responsiveness in mutant lines

Besides a negative influence on morphogenesis, Gr suppression is clearly associated in mutants with unbalancing of fundamental regulatory pathways. This is exemplified by the up-regulation along the HPI axis, leading to increased basal levels of *crh* and *pomca* transcripts as well as cortisol content in *gr*^−/−^ larvae. Corticosteroidogenesis was apparently supported also by increased *star* gene expression and shifting from cortisone to cortisol by down-regulation of *11β*-*hsd type 2* gene. This scenario underscores the failure of the negative feedback loop mediated by the GC-Gr complex despite the increased cortisol concentration.

The overstimulation of basal HPI axis was also associated with unresponsiveness to a prolonged mechanical stressor, denoting that the HPI axis was completely disrupted and deprived of function. Also the *gr*^*s357*/*s357*^ strain showed comparable signs of axis perturbation, such as higher levels of cortisol concentration and basal *pomca* expression. In this line, basal *crh* gene expression was higher than in WT, though not significantly. Whereas *pomca* transcription is primarily down-regulated by GCs through nGRE^39^, repression of *crh* seems to involve also protein-protein interactions^40^ and thus to be at least partially active in this mutant line. Interestingly, up-regulation of basal *crh* and *pomca* transcription was observed in larvae born from eggs with antibody-sequestered cortisol, while unresponsiveness to an acute physical stressor, in terms of cortisol level, was found in larvae born from eggs loaded with cortisol. In both cases, *gr* gene expression was unchanged. These effects were interpreted as evidence of maternal cortisol programming of post-hatch HPI axis activity^41^.

### Derangement of immune responses in the *gr*^−/−^ mutant line

A key point is that defence mechanisms in the *gr*^−/−^ strain were hampered not only at the level of the stress response, usually to cope with predators and competitors, but also at the level of the immune response to counteract pathogens and noxious substances. In agreement with results obtained by Chatzopoulou and coworkers^2^ with a tail fin amputation assay, the challenge with DSS induced a pro-inflammatory reaction with enhanced transactivation of the genes encoding the cytokines Il1β, Il8 and Il6 and the metalloproteinase Mmp-13, involved in inflammatory processes, in both WT and *gr*^s357/s357^ larvae, but not in the *gr*^−/−^ strain. Subsequent treatment with DEX significantly depressed the transcription of the above genes, whereas no change was observed in the *gr*^−/−^ strain. This is more clearly illustrated by the absence of DEX repression of *mmp*-*9* gene expression without DSS challenge, as imputed above to missing protein-protein interaction in the *gr*^−/−^ strain. Cross talk between GR and proinflammatory transcription factors normally controls the down-regulation of pro-inflammatory cytokines^42,43^.

These findings are in keeping with the dual-action model of the GC-GR complex in the immune response, proposed by Busillo and Cidlowski^21^ and Duque and Munhoz^44^. Accordingly, in the initial phase of acute inflammatory response, the complex would exert pro-inflammatory effects, relying on innate immunity and the up-regulation of tumor necrosis factor alpha that leads to enhanced cytokine expression^45^. In the following recovery phase, the complex would manifest its well-known anti-inflammatory potency. Thus, the *gr*^−/−^ line appears as a good model when compared to WT and *gr*^s357/s357^ to probe the complex transcriptional interplay in the phasing of the inflammation response.

The *gr*^−/−^ line characterization suggests two considerations. Firstly, a less dissipative metabolism for low stress responsiveness is a selected trait in animal breeding, but is often linked to weak immune competence and propensity for fattening^46^. Secondly, in zebrafish, as in other teleosts, Gr is evolutionarily entangled with the mineralocorticoid receptor (Mr), since cortisol is a common Mr/Gr ligand^47^. Thus, it will be important to analyse GC functions also in relation to this receptor, which shares with Gr the hormone responsive elements as well.

In conclusion, the *gr*^−/−^ line represents a significance advance with respect to previous zebrafish mutant lines, as it allows more extensive silencing of Gr regulatory cascade that can be profitably confronted with those in normal and partially Gr-silenced fish. Moreover, the *gr*^−/−^ line provides a convenient platform to generate a Gr/Mr-double null line and appears as a promising tool for research in fast-advancing fields on Gr actions, like morphogenesis, maternal developmental programming and epigenesis, obesity and intermediary metabolism dysfunctions, immune deficiency and hypertension.

## Methods

### Zebrafish maintenance

Wild type and mutant zebrafish were staged and maintained according to standard procedures^48^. Embryos were obtained by natural mating and raised at 28 °C in Petri dishes containing fish water (50X: 25 g Instant Ocean, 39.25 g CaSO_4_ and 5 g NaHCO_3_ for 1 l) and kept in a 12:12 light-dark (LD) cycle. For screening and *in vivo* imaging, embryos and larvae were anesthetized with 0.04% tricaine (Sigma-Aldrich, E10521). The *gr*^*s357*^ mutant line, kindly provided by Prof. Herwig Baier (Max Planck Institute of Neurobiology, Germany), as well as the *gr*^−/−^ line (this work) were prepared on Tg(9xGCRE-HSV.Ul23:EGFP)^ia20^ background^27^. In all experiments controls (*gr*^+/+^) and mutant larvae derive from different batches of eggs. All larvae were sampled at the same time of the day (between 15:00–17:00 h) to reduce the possibility of fluctuations due to diurnal circadian cortisol production.

All husbandry and experimental procedures complied with European Legislation for the Protection of Animals used for Scientific Purposes (Directive 2010/63/EU). The experimental protocol was previously authorized by the University of Padova, Body for the Protection of Animals (OPBA-Project Number 112/2015).

### Generation of the *gr* mutant line

*Gr* mutant cells were generated using the CRISPR/Cas9-mediated genome editing. Gene-specific guide RNA (sgRNA) was designed against an optimal CRISPR site in exon 2 of *gr* (NM EF567112.1) using the CHOPCHOP software (available at: https://chopchop.rc.fas.harvard.edu). *Gr* target sequence and CRISPR oligonucleotides are listed in Table S1. BLAST analysis of the target sequence revealed no specific binding with other genes. sgRNA was generated according to previously described methods^49^ and *in vitro* transcribed using MEGAshortscript T7 kit (Life Technologies, AM1354). Cas9 mRNA was transcribed from linearized pCS2-nls-zCas9-nls plasmid (Addgene, Plasmid #47929) using mMessage Machine SP6 kit (Life Technologies, AM1340).

Fertilized eggs were injected with 1 nl of a solution containing 400 ng/μl Cas9 mRNA and 50 ng/μl gRNA. Genomic DNA was extracted from 5-dpf larvae from individually injected eggs to verify the presence of mutations and confirm the activity of the guide RNA. Injected embryos were raised to adulthood and F0 founders selected by genotyping (see below). Embryos collected from the outcrosses between these F0 founders and WT were raised and genotyped to confirm germline transmission of the mutation (F1 generation). Heterozygous mutants with the same mutation were selected and crossed, to obtain homozygous mutant embryos (F2 generation).

### Genotyping *gr*^−/−^ mutants

Larvae or adult fish were anesthetized in tricaine and a small fragment of the caudal fin was cut with a sharp blade. Genomic DNA was extracted using the HotSHOT protocol^50^.

Mutations in F0 were detected using heteroduplex mobility assay (HMA)^51^. In this case, genomic fragments at the target sites were amplified by PCR with 5x HOT FIREPol® Blend Master Mix (Solis BioDyne, 04-25-00125) and the locus-specific primers (*gr*-F1 and *gr*-R1) listed in Table S1.

PCR conditions were as follows: 15 min at 95 °C, 35 cycles at 95 °C for 20 s, 60 °C for 30 s and 72 °C for 30 s. The resultant PCR amplicons were electrophoresed on a 15% polyacrylamide gel (Life Technologies, NP0323PK2). For verification, PCR products from fish harbouring indel mutations were subjected to sequencing. Poly Peak Parser software (http://yosttools.genetics.utah.edu/PolyPeakParser/) was used for identification and sequence characterization of heterozygous mutant carriers generated by genome editing^52^.

Screening primers for heterozygous and homozygous fish, *gr*-F2 and *gr*-R2 (see Table S1 and Supplemental Fig. 1, panel C), were designed to amplify a 82-bp region across the *gr* sgRNA target region. PCR products were resolved with ethidium bromide-stained 3% agarose low EEO gel (Fisher BioReagents, BP160-500) in order to identify *gr*^+/+^, *gr*^+/−^ and *gr*^−/−^ samples.

*gr*^*s357*/*s357*^ mutants were identified by sequencing of a 223-bp fragment which includes the *gr*^*s357*^ point mutation obtained by genomic DNA PCR amplification with primers s357-F1 and s357-R1 (Table S1).

### Sorting *gr*^−/−^ and *gr*^s357/s357^ mutants by VBA assay

To identify homozygous mutants obtained by crossing F1 heterozygous *gr*^+/−^ or *gr*^*s357*/*+*^ mutants, larvae were subjected to the VBA assay at 96 or 120 hpf.

After 30 min of dark adaptation in an opaque plastic box, larvae were exposed for 20 min to a white background under bright, whole-field illumination (using a 30 W fluorescent lamp mounted 45 cm above the dish) and analysed for lack of melanophore contraction^23^.

### Treatments of zebrafish larvae

For steroid hormone treatment, groups of 4-dpf larvae (n = 15) of each zebrafish condition (*gr*^−/−^, *gr*^*s357*/*s357*^ and *gr*^+/+^) in ia20 background were incubated for 24 h in fish water containing either 10 μM dexamethasone (DEX) (Sigma, D1756) or vehicle alone (ethanol). The following day, larvae were analysed at the fluorescence microscopy and used for total RNA extraction.

For mechanical stress induction, groups of 5-dpf larvae (n = 15) of each zebrafish condition (*gr*^−/−^, *gr*^*s357*/*s357*^ and *gr*^+/+^) were placed in 20 ml of fish water on 50-ml beaker over a magnetic stirrer and agitated for 20 min at the speed of 300 rpm^53^. Control and experimental larvae were then euthanized, frozen in liquid nitrogen and stored at −80 °C until processed for whole-embryo cortisol analysis or RNA extraction.

To induce intestinal inflammation with dextran sodium sulphate (DSS, 40,000 MW, Sigma, 42867), a 0.5% (w/v) working dose was prepared in fish water, starting from a 10% (w/v) stock concentration, freshly made to avoid loss of DSS solution quality due to decomposition of the product^30^. Inflammation was induced in 3-dpf larvae of each condition (*gr*^−/−^, *gr*^*s357*/*s357*^ and *gr*^+/+^) exposed to DSS by immersion for 20 h. After DSS removal, half of the larvae were then exposed to DEX for 24 h and the other in regular fish water. Control larvae were maintained in regular fish water. Each treatment was performed four times with 15 larvae per replica.

### Steroid measurements in zebrafish gr mutant larvae

Steroid hormones were extracted and whole-embryo cortisol measured by radioimmunoassay (RIA), as described by Bertotto and coworkers^54^. The analyses were performed at the Department of Comparative Biomedicine and Food Science, University of Padova, with anti-cortisol serum (Analytical Antibodies, Bologna). Cortisol measurements are expressed in pg/µg protein. Protein concentration was determined by BCA protein assay kit (Thermo Fisher Scientific, 23227).

### RNA isolation and quantitative real time reverse transcription PCR (qRT-PCR)

For expression analysis, total RNA was extracted from pools of 15 larvae with TRIzol reagent (Thermo Fisher Scientific, 15596026). Poly(A) mRNA was purified from 5 μg of total RNA with the Dynabeads “mRNA direct kit” (Thermo Fisher Scientific, 61011) and used for cDNA synthesis with M-MLV Reverse Transcriptase RNase H- (Solis BioDyne, 06-21-010000) according to the manufacturer’s protocol. PCRs were performed with the SYBR green (Bio-Rad Laboratories) method in a CFX96™ Real-Time PCR thermal cycler (Bio-Rad Laboratories). *ribosomal protein L13a* (*rpl13a*) and *ribosomal protein*, *large*, *P0* (*rplp0*) were used as internal standards in each sample in order to standardize the results by eliminating variation in mRNA and cDNA quantity and quality. The annealing temperature for PCR ranges from 58 to 60 °C, depending on the primer set used. The cycling parameters were 95 °C for 10 min, followed by 45 cycles at 95 °C for 30 s and annealing-extension for 30 s. No amplification products were observed in negative controls and no primer-dimer formations in the control templates. The data obtained were analysed using the iQ5 optical system software version 2.0 (Bio-Rad) including GeneEx Macro iQ5 Conversion and Genex Macro iQ5 files. All analyses were performed in triplicate. Primer sequences are reported in Table S1.

### Morphological and histological analysis

*gr*^−/−^, *gr*^*s357*/*s357*^ and *gr*^+/+^ 45-dpf old and *gr*^−/−^ and *gr*^+/+^ 8-mpf old zebrafish were fixed for 24 h in Bouin’s solution at room temperature. The samples were dehydrated through a graded series of ethanol and embedded in Paraplast plus (Leica, 39602004). The samples were serially cut into 7/8-μm sections on a LKB microtome. After rehydration, the sections were stained with haematoxylin and eosin and mounted with Eukitt (BioOptica, 09-00100) for microscopic examination.

### Alcian blue staining

Larvae subjected to the intestinal inflammation treatments were fixed in 4% paraformaldehyde, rinsed in acidic ethanol and stained with Alcian blue as described^30^. Unbound stain was removed by repeated rinsing with acidic ethanol prior to whole-mount imaging.

### Heart rate

Heart rates were determined by counting the number of atrial contractions during 15 s in embryos at 5 dpf using slow-motion replay of videotape recordings.

### Imaging

For imaging, transgenic embryos and larvae were embedded in 1% low-melting agarose. Fluorescence was visualized at the Leica M165FC dissecting microscope and photographed with a Leica DFC7000 digital camera. All images were analyzed with Fiji software^55^.

### Western blotting

Total protein extracts were obtained by homogenizing livers from adult zebrafish (*gr*^+/+^ and *gr*^−/−^) in ice-cold RIPA buffer (125 mM NaCl, 25 mM Tris-HCl pH 7.4, 1 mM EGTA-TRIS pH 7.4, 1% Triton-X100, 0.5% sodium deoxycholate, 0.1% SDS) and Complete EDTA-free protease inhibitor cocktail (Sigma, 11873580001) on ice. For Western blot analysis 50 μg of protein extracts were loaded per lane on 4–12% Bis-Tris NuPage gels (Thermo Fisher Scientific, NW00102BOX) SDS/PAGE and blotted on PVDF Immobilon-P membranes (Millipore, IPFL00010). Dried membranes were then washed with TBS buffer (50 mM Tris–HCl pH 7.5, 50 mM NaCl) with 1% (w/v) bovine serum albumin (BSA, Sigma, A2153) and incubated overnight with the indicated primary antibodies at 4 °C: anti-GR (1:500; Santa Cruz Biotechnology, sc-1002;); anti-Tom20 (1:5000; Santa Cruz Biotechnology, sc-11415). Secondary anti-Rabbit HRP-conjugated antibody (BIORAD, 1706515) were incubated for 1 h at room temperature and protein bands detected by chemiluminescence on an Alliance MINI HD 9 Western Blot Imaging System. Quantitation of the signal was performed with ImageJ.

### Statistical analysis

Statistical analysis was performed using Graph Pad Prism V6.0. Data are presented as the means ± SEM. Statistical analysis of comparison between WT and mutant fish lines was performed with two-way ANOVA followed by Tukey’s multiple comparison test, except for experiments showed in Fig. 5, Supplemental Fig. 1 and Supplemental Fig. 4 in which one-way ANOVA was used. The P values are summarized with the following symbols: *P < 0.05, **P < 0.01, ***P < 0.001 or different letters (significance was set at *P* < 0.05). Survival was analysed using Kaplan–Meier survival curves, and statistical significance was verified via the log-rank test.

## Electronic supplementary material

Supplementary figures

Supplementary Table
