# Author Correction: *nr3c1* null mutant zebrafish are viable and reveal DNA-binding-independent activities of the glucocorticoid receptor

**DOI:** 10.1038/s41598-018-21745-8

**Published:** 2018-03-08

**Authors:** N. Facchinello, T. Skobo, G. Meneghetti, E. Colletti, A. Dinarello, N. Tiso, R. Costa, G. Gioacchini, O. Carnevali, F. Argenton, L. Colombo, L. Dalla Valle

**Affiliations:** 1 Department of Biology, University of Padova, Padova, Italy; 2 Department of Molecular Medicine, University of Padova, Padova, Italy; 3 Department of Life and Environmental Sciences, Marche Polytechnic University, Ancona, Italy

Correction to: *Scientific Reports* 10.1038/s41598-017-04535-6, published online 29 June 2017

In the original version of this Article, the authors neglected to cite a previously-published paper which reports prior *in vitro* analysis of the transcriptional activity of *grs*^357/s357^ mutant line. As a result, in the Discussion section under subheading ‘**Divergence in GRE-independent target gene transcription in the two mutant lines**’.

“Divergent transcriptomic responses were observed with DEX suppression of the DSS-induced transcription of the cytokine genes *il1β, il8* and *il6* and of the metalloproteinase genes *mmp-13* and DSS-unchallenged *mmp-9* in *grs*^357/s357^ mutants, but not in the other ones. Hence, the *grs*^357/s357^ line directly reveals GRE-independent GC-GR transcribing activities due to their persistence, while the *gr*^−/−^ line indirectly confirms their occurrence due to their absence with consequent effects on the phenotype, as discussed in details below.”

Now reads:

“Divergent transcriptomic responses were observed with DEX suppression of the DSS-induced transcription of the cytokine genes *il1β, il8* and *il6* and of the metalloproteinase genes *mmp-13* and DSS-unchallenged *mmp-9* in *grs*^*357/s357*^ mutants, but not in the other ones. Transcriptional activity of *grs*^*357/s357*^ mutant line was *in vitro* analysed by Ziv and co-workers (2013)^31^ that found lack of Gr genomic activity including transcriptional repression linked to AP1 or NF-kB transcription factors. However, these data are not in conflict with our definition of *grs*^*357/s357*^ as being partially silenced: Gr can regulate gene repression either by binding directly to a GRE and interacting with other transcription factors^32^ (and this way is lost also in *grs*^*357/s357*^) or by tethering with other DNA binding proteins, a way lost in the *gr*^−/−^ null line while possibly retained in the *grs*^*357/s357*^ mutant. Hence, the *grs*^*357/s357*^ line directly reveals GRE-independent GC-GR transcribing activities due to their persistence, while the *gr*^−/−^ line indirectly confirms their occurrence due to their absence with consequent effects on the phenotype, as discussed in details below.”

Additionally, a supplementary table containing the primers used was omitted from the original version of this Article. These errors have been corrected in the HTML version and the PDF version of the Article.

