# Supplementary material for: nr3c1 null mutant zebrafish are viable and reveal DNA-binding-independent activities of the glucocorticoid receptor
Source: Sci Rep. 2017 Jun 29;7:4371. doi: 10.1038/s41598-017-04535-6 (PMC5491532; doi:10.1038/s41598-017-04535-6)
Supplement: Supplementary file 1 — Supplementary figures [file 41598_2017_4535_MOESM1_ESM.pdf]

nr3c1 null mutant zebrafish are viable and reveal DNA-binding-independent activities of the glucocorticoid receptor

[1]Facchinello N, [1]Skobo T, [2]Meneghetti G, [1]Colletti E, [1]Dinarello A, [1]Tiso N, [2]Costa R, [3]Gioacchini G., [3]Carnevali O, [1]Argenton F, [1]Colombo L, [1]Dalla Valle L.

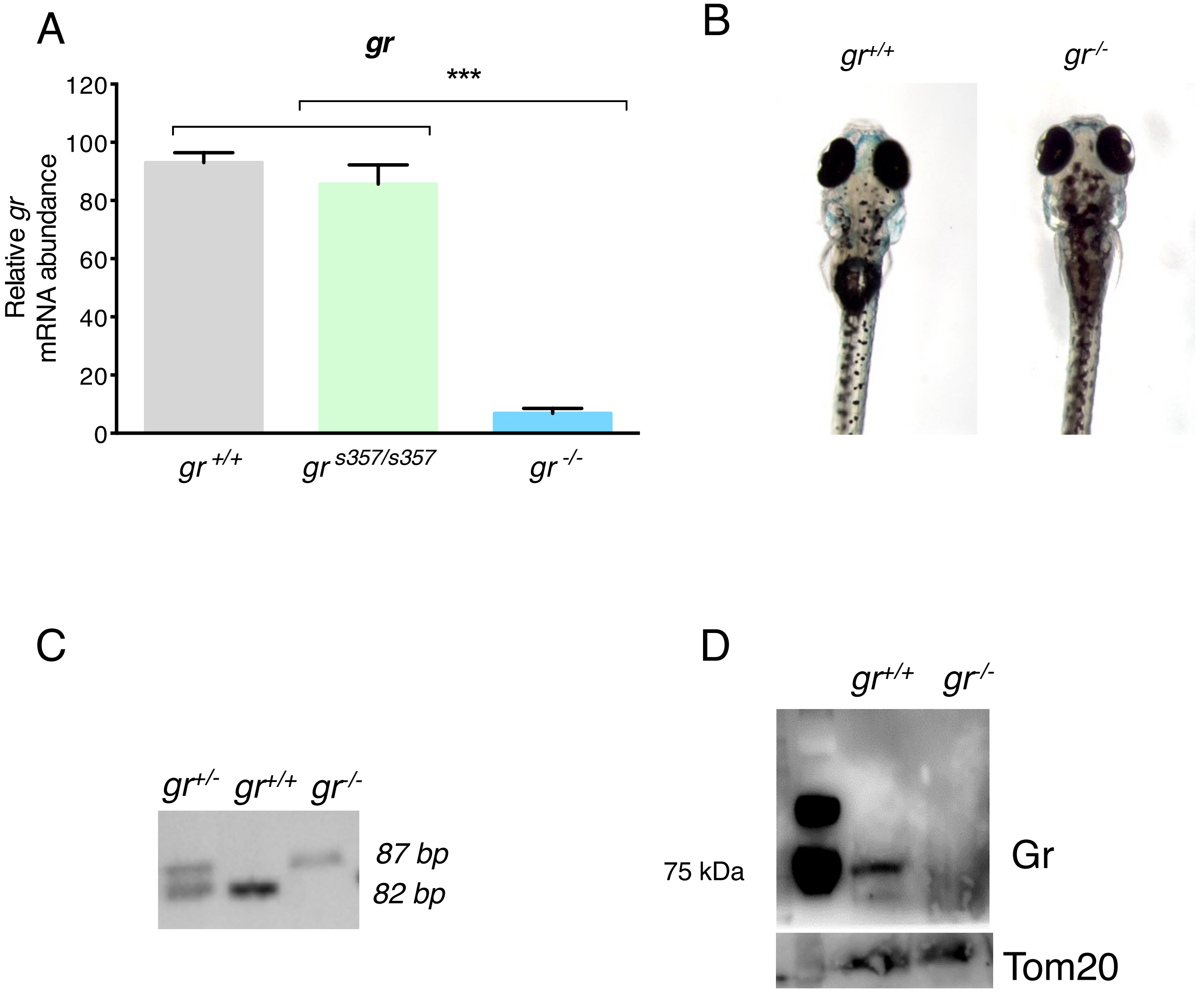

Supplemental Fig. 1. (A): qRT-PCR of *gr* mRNA in 5-dpf mutant larvae compared to control shows a statistically significant reduction of *gr* expression. Values represent the mean ± SEM. Asterisks indicate that expression levels are significantly different from the control: ***P < 0.001. Data were generated from four biological replicates.
(B): Representative images of 5-dpf control $gr^{+/+}$ and mutant $gr^{-/-}$ larvae after exposure to VBA stimulus. $gr^{-/-}$ mutants appear darker in comparison to control. (C): Representative gel image of PCR genotyping using genomic DNA from tail fins of adults born from a cross between *gr* heterozygotes. (D): Western blot of liver proteins from 8-month-old $gr^{-/-}$ and $gr^{+/+}$ zebrafish showing disappearance of the protein band with respect to control.

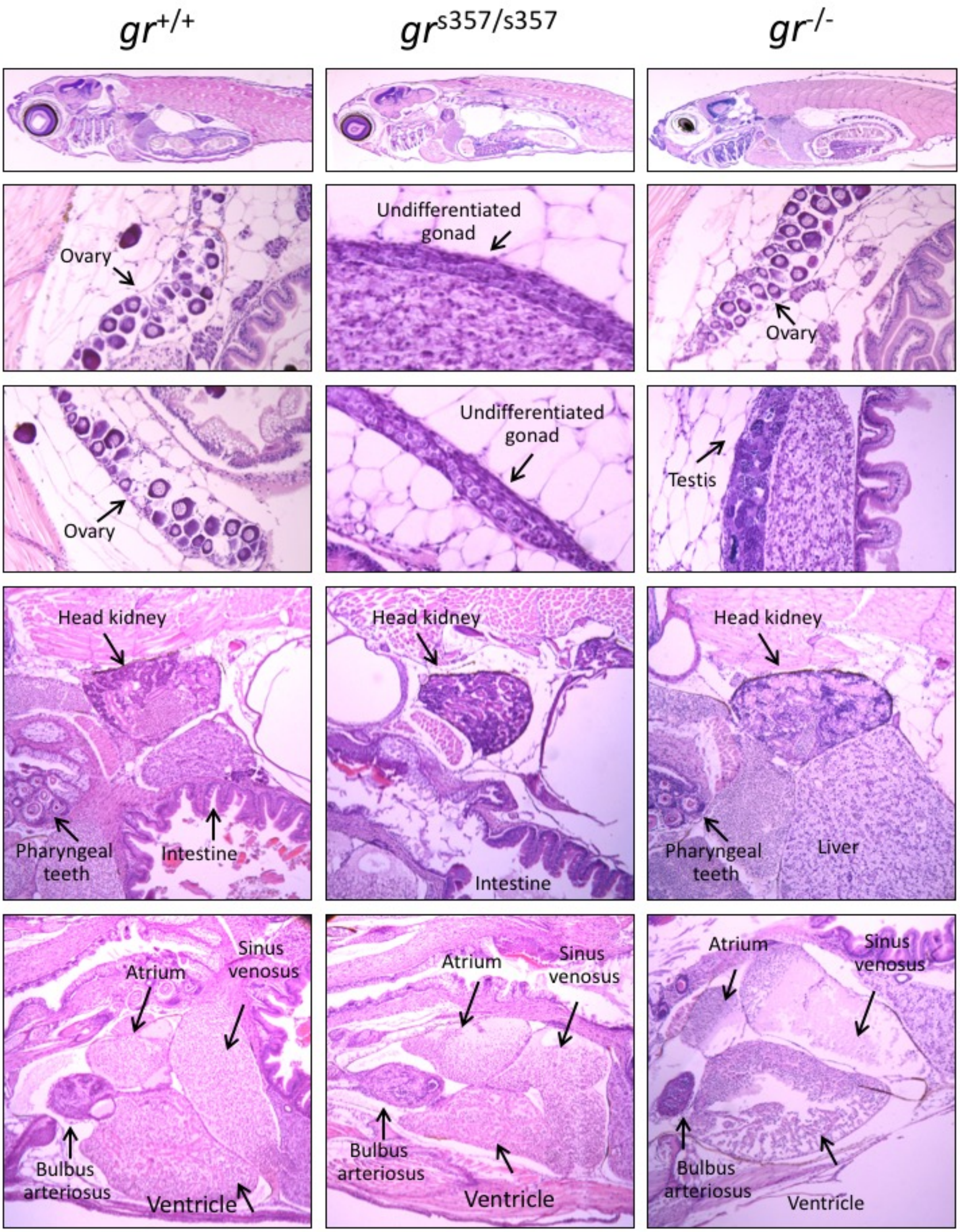

Supplemental Fig. 2a. Histological analysis of two samples of each genotype, *gr*$^{+/+}$, *gr*$^{s357/s357}$ and *gr*$^{-/-}$ zebrafish, at 45 dpf of age. All histological images were taken from longitudinal sections stained with haematoxylin and eosin (H&E). Panels compare tissues and structures in the three different genotypes. Top 3 panels present a total body section of one sample of each genotype. Middle 9 panels present details of the gonads, head kidneys and pharyngeal teeth. Bottom 3 panels show a longitudinal section of the heart showing the reduced trabecular network of the *gr*$^{-/-}$ heart ventricle.

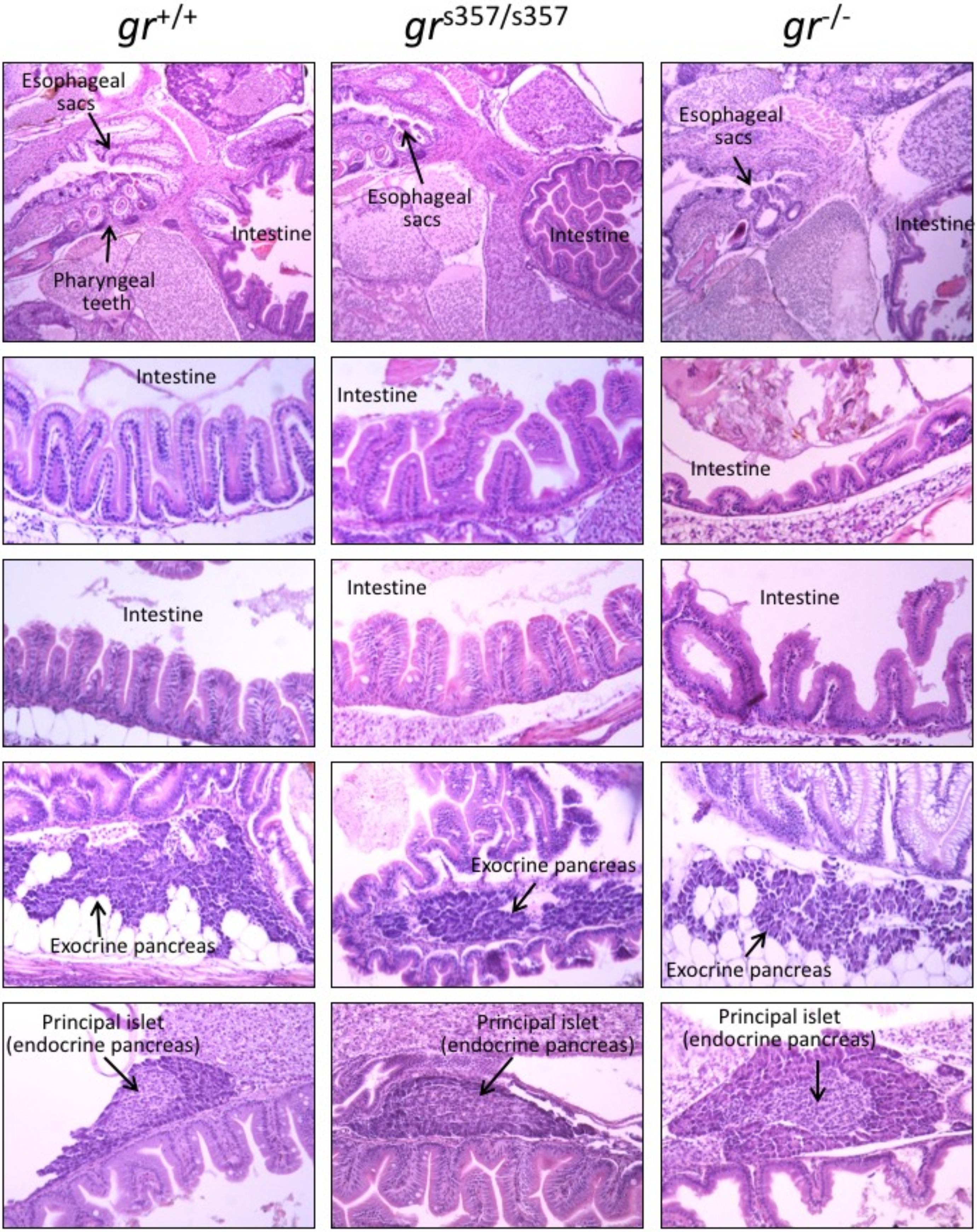

Supplemental Fig. 2b. Histological analysis of two samples of each genotype, $gr^{+/+}$, $gr^{s357/s357}$ and $gr^{-/-}$ zebrafish, at 45 dpf of age. All histological images were taken from longitudinal sections stained with haematoxylin and eosin (H&E). Top 3 panels present sections of esophageal sacs and proximal intestine. Middle 6 panels present details of the intestine showing the presence of a thinner epithelium in $gr^{-/-}$ samples. Bottom 6 panels show details of the endocrine and exocrine pancreas.

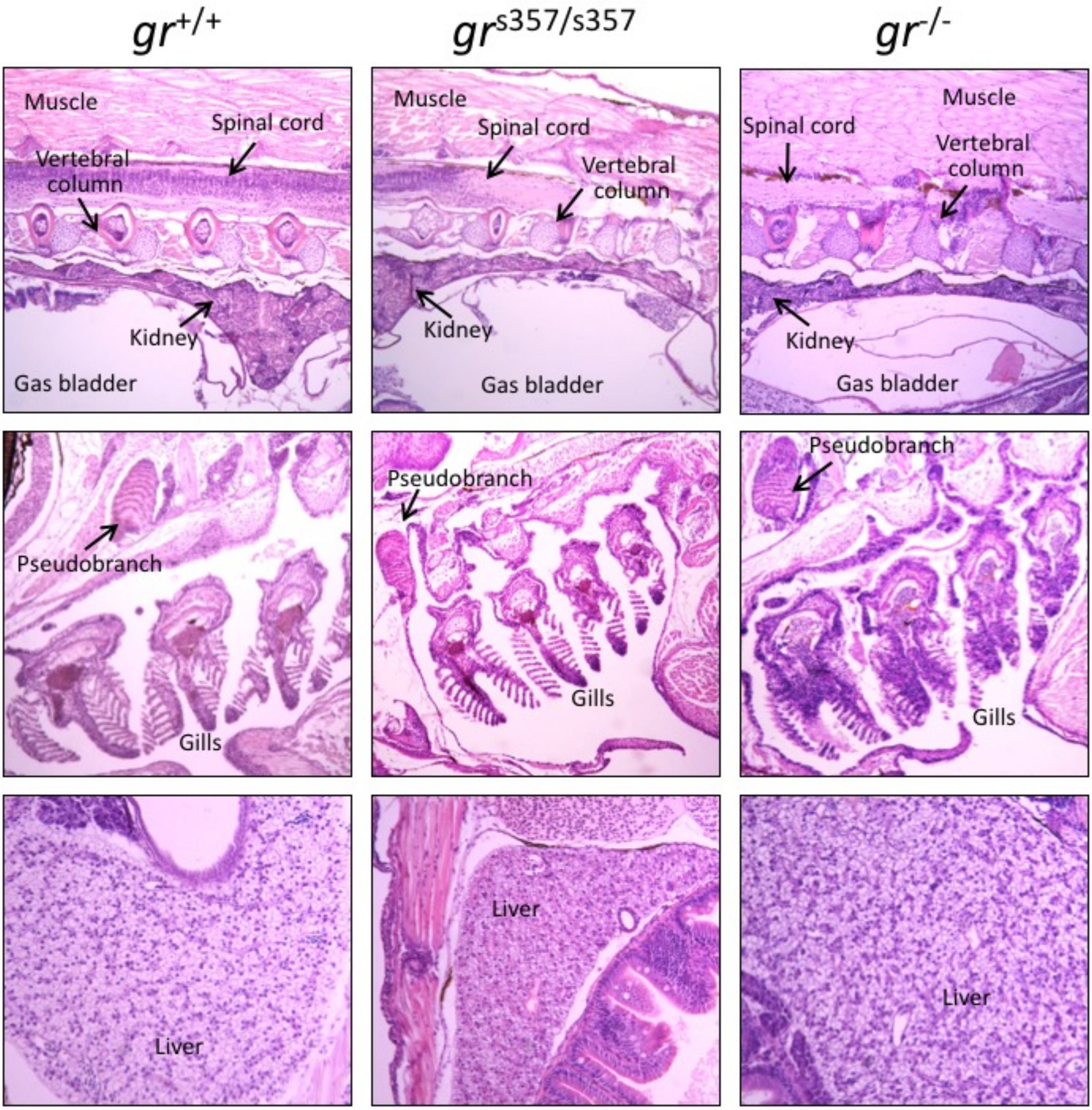

Supplemental Fig. 2c. Histological analysis of two samples of each genotype, *gr*$^{+/+}$, *gr*$^{s357/s357}$ and *gr*$^{-/-}$ zebrafish, at 45 dpf of age. All histological images were taken from longitudinal sections stained with haematoxylin and eosin (H&E). Top 3 panels present sections of spinal cord and vertebral column together with the kidney. Middle 3 panels present details of pseudobranch and gills. Bottom 3 panels show sections of the liver.

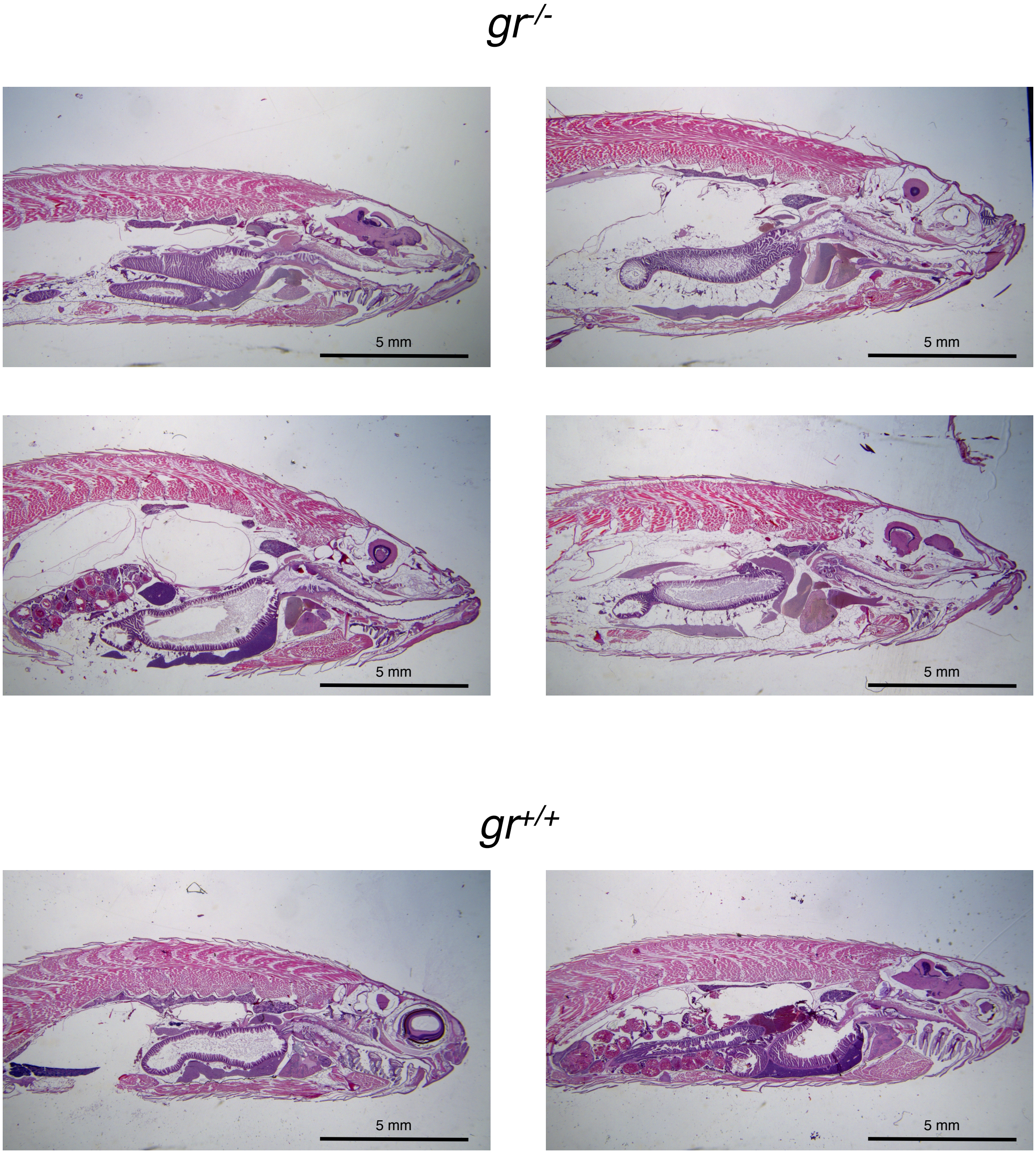

Supplemental Fig. 3. Longitudinal sections stained with haematoxylin and eosin (H&E) of four *gr*$^{-/-}$ and two *gr*$^{+/+}$ to better visualize the adipose tissue increase in mutants.

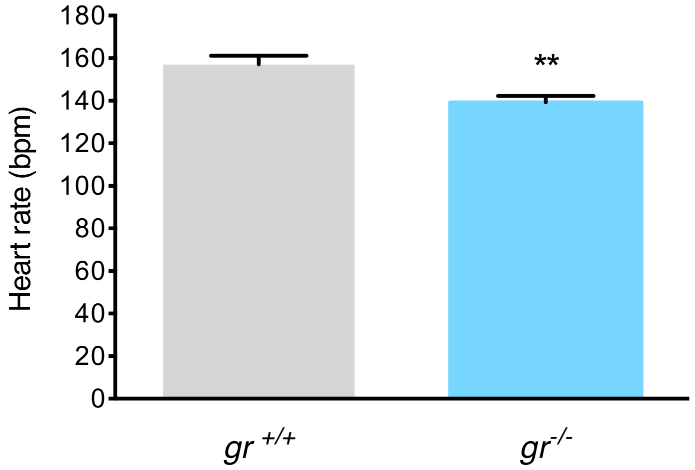

Supplemental Fig. 4. Decrease of heart rate in 5-dpf *gr*$^{-/-}$ with respect to *gr*$^{+/+}$. n = 12. Asterisks indicate that the heart rate is significantly different from the control (one-way-ANOVA, **P < 0.01).

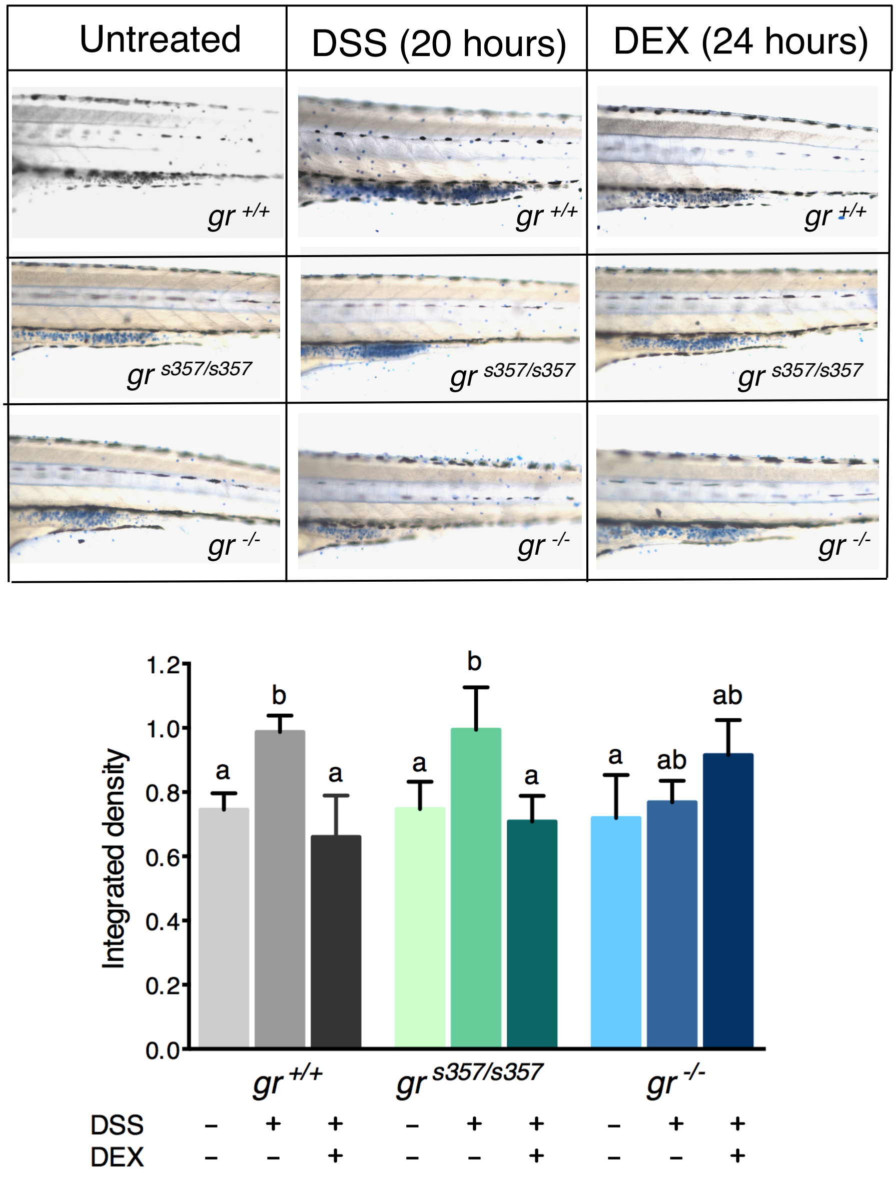

Supplemental Fig. 5. (A): Whole-mounts of the posterior intestinal region of control, DSS-exposed and DSS plus DEX-exposed larvae of the three genotypes analysed after staining with alcian blue. (B): Comparison of alcian blue-stained mucous granules in the intestine of the above larvae. n = 15 larvae for each group. Values represent the mean ± SEM. Different letters indicate statistically significant differences checked by two-way ANOVA followed by Fisher's post hoc test ($p<0.05$).
