# Supplementary material for: nr3c1 null mutant zebrafish are viable and reveal DNA-binding-independent activities of the glucocorticoid receptor
Source: Sci Rep. 2017 Jun 29;7:4371. doi: 10.1038/s41598-017-04535-6 (PMC5491532; doi:10.1038/s41598-017-04535-6)
Supplement: Supplementary file 2 — Supplementary Table [file 41598_2017_4535_MOESM2_ESM.pdf]

Supplementary Table 1

CRISPR target site oligonucleotides (Gagnon et al., 2014)

Oligo name	Sequence
Constant oligo	AAAAGCACCGACTCGGTGCCACTTTTTCAAGTTGATAACGGACTAGCCTTATTTTAACTTGCTATTTCTAGCTCTAAAAC
Gr specific oligo	ATTTAGGTGACACTATA***GGCAGACCGACGACAGCTGT*G**TTTTAGAGCTAGAAATAGCAAG

*Bold and italic sequence correspond to gRNA

PCR primers used in mutation screens for *gr*

Gene	Accession		Primer (5′–3′)
$gr^{-/-}$	NM_001020711	F1	GCTCTCCTTTCAGAGCTGCC
		R1	CTCTGCTGCATTCCACTGAC
		F2	ACCACTTCAAGCGGACAGAG
		R2	CCGGCTTCTGATCTTTCTGC
$gr^{s35/s3577/s357}$	NM_001020711	F1	GTCTCTTGACACATCCTG
		R1	CTGACATTTAAGGACACACTG

PCR primers used in for qRT-PCR

Gene	Accession		Primer (5′–3′)
rplp0 (arp)	NM_131580	F	CTGAACATCTCGCCCTTCTC
		R	TAGCCGATCTGCAGACACAC
rpl13a	NM_212784	F	TCTGGAGGACTGTAAGAGGTATGC
		R	AGACGCACAATCTTGAGAGCAG
gr	NM_001020711	F	GACAGCACTATACCAGACAC
		R	CTTCAACATCTGTTCACAC
star	NM_131663	F	TGTAAGGGCTGAGAATGG
		R	TCAGCAAGCAATGGCTGC
11β-hsd2	NM_212720	F	GTCCTCTGTGTGTGCTGC
		R	GCTTGCTGTACCTGCTGAG
crh	NM_001007379	F	GAATGTAGAGCCATCGAGAGC
		R	CCCTCCAACAGACGCTGC
pomca	NM_181438	F	TGTCGAGACCTCAGCACAG
		R	TGCGAGGAGGTCGATTTGC
fkbp5	NM_213149	F	GTGTTCGTCCACTACACC
		R	TCTCCTCACGATCCCACC
Il1β	NM_212844	F	GACATGCTCATGGCGAACG
		R	GCAAATCGTGCATTGCAAGACG
Il6	NM_001261449	F	GTGAAGACACTCAGAGACG
		R	GTTAGACATCTTTCCGTGCTG
Il8	XM_009306855	F	TGTTTTCCTGGCATTTCTGACC
		R	TTTACAGTGTGGGCTTGGAGGGG
mmp-9	NM_213123	F	CATTAAAGATGCCCTGATGTATCCC
		R	AGTGGTGGTCCGTGGTTGAG
mmp-13	NM_001290479	F	ATGGTGCAAGGCTATCCCAAGAGT
		R	GCCTGTTGTTGGAGCCAAACTCAA
